# When is Meaning in Life Most Beneficial to Young People? Styles of Meaning in Life and Well-Being Among Late Adolescents

**DOI:** 10.1007/s10804-017-9280-y

**Published:** 2017-11-30

**Authors:** Dariusz Krok

**Affiliations:** Institute of Family Sciences, Opole University, ul. Drzymały 1a, 35-342 Opole, Poland

**Keywords:** Meaning in life, Late adolescence, Subjective well-being, Psychological well-being

## Abstract

The purpose of this article was to investigate the relationships between different dimensions of meaning in life and subjective and psychological well-being (PWB) among late adolescents. Three hundred and eighty four Polish participants completed The Personal Meaning Profile scale, The Satisfaction With Life Scale, The Positive and Negative Affect Schedule, The PWB scale, and the Meaning in Life Questionnaire. Two studies were conducted. In Study 1, the findings revealed that personal meaning had strong relations with subjective well-being (SWB) and PWB. In addition, the dimensions of personal meaning were more strongly associated with the cognitive dimension of SWB than with PWB. In Study 2, search for meaning had positive associations with SWB and PWB among those late adolescents who already had substantial meaning in life. Individuals who were in presence and search style had higher levels of SWB and PWB than those in only search style or presence style. The results demonstrate that purpose embedded in the concept of meaning in life appears central to the formation of adolescent well-being as young people come to establish overarching aims.

## Introduction

The scientific study of meaning has largely concentrated on recognizing the consequences of believing that one’s life is meaningful (Głaz 2013; Klinger 2012; Steger 2012). In this sense, meaning in life is regarded as a factor that makes a unique contribution to the sphere of well-being and happiness. Meaning in life has unique psychological assets that influence human cognitive and emotional processes in late adolescence. During this developmental period teenagers typically strive to achieve an integrated view of themselves, including their own pattern of beliefs, goals, and motivations (Boyd and Bee 2012; Brassai et al. 2011). Yet, very little research has been conducted on the psychological associations of search for meaning in life among late adolescents. It would be beneficial to gain more knowledge of the characteristics of presence and search mechanisms, and whether young people with high levels of meaning in life who are nevertheless searching for meaning can obtain higher subjective and psychological well-being (PWB). The present study extends this work by examining to what extent search for meaning is a significant factor for young people’s well-being.

## Meaning in Life in Late Adolescence

Frankl (1965) defined meaning in life in terms of the will-to-meaning which was conceptualized as the innate desire to give as much meaning as possible to an individual’s life and to actualize as many values as possible. He considered the will-to-meaning to be an essential human motive. Meaning in life can be conceptualized as an individually constructed cognitive system, which endows life with personal significance (Wong 1989). The operationalization views meaning in life as a cognitive personality trait that is primarily rooted in human cognition and differs between individuals. It motivates people to discover significant aspects of their lives and serves vital functions in the process of goal striving.

Research conducted by Steger (2009), Steger and Frazier (2005), Steger et al. (2006) demonstrated that meaning in life can be viewed in two main dimensions: presence of meaning and search for meaning. The former concerns the degree to which individuals perceive their lives as significant and meaningful, while the latter reflects the degree to which people are engaged in a search for meaning in life. Both dimensions have an essentially different character and express dissimilar attitudes to life (Steger 2009). Presence of meaning predisposes people to experience their lives as comprehensible and significant, and to feel a sense of purpose or mission in their daily endeavours. Search for meaning represents the dynamic and active efforts individuals expend when they try to comprehend the meaning, significance, and purpose of their lives.

Meaning in life plays an important role in late adolescence. While facing new situations and events, young people try to interpret and organize their experience by identifying significant aspects of their personal and social life, and discovering deeper meanings in their lives (Kiang and Fuligni 2010; Reker 2005). The presence and search dimensions may play a different role in adolescents’ lives as they actively try to deepen their understanding of significance and purpose of themselves and their lives. From the life-span perspective, young people show distinctive characteristics related to interpreting and organizing their experience, pursuing important goals, and making sense of the world and themselves (Boyd and Bee 2012; Dezutter et al. 2014). Search for meaning in life may have a positive function as a cognitive schema that enables late adolescents to identify and interpret information relevant to meaning in life judgments and to integrate their life experiences.

## Relationships Between Meaning in Life and Subjective and Psychological Well-Being

These questions are central to the science of meaning and well-being, especially as research indicates that search for meaning is associated with higher neuroticism, negative affect, anxiety, and depression (Debats et al. 1993; Steger et al. 2006). Yet, search for meaning in life, which refers to people’s desire and efforts to enhance their understanding of the meaning, significance, and purpose of their lives, does not necessarily have to be disadvantageous and harmful to well-being. Under certain conditions, it might lead to positive outcomes.

Meaning in life has played an important role in research on well-being and quality of life in recent years (Park et al. 2010; Steger 2009; Wong 2012). Well-being is usually divided into two types: subjective well-being (SWB) and PWB, which are based on different conceptual traditions.

SWB is defined as “a person’s cognitive and affective evaluations of his or her life as a whole” (Diener et al. 2009, p. 187). Being based on a hedonic approach, it encompasses cognitive judgments of satisfaction and fulfilment as well as emotional reactions to events. SWB has three distinct components: life satisfaction, positive affect, and negative affect. PWB is based on an eudaimonic approach that perceives life in terms of virtues and self-realization (Ryff 1989; Ryff and Singer 2008). According to Ryff (1989), this type of well-being includes six dimensions: (1) self-acceptance, (2) positive relations with others, (3) autonomy, (4) environmental mastery, (5) purpose in life, and (6) personal growth. Taken together, they represent an integrative vision of human life.

There is empirical evidence that meaning in life contributes to SWB. The results of several studies have demonstrated that meaning in life was positively linked to life satisfaction (Zika and Chamberlain 1992) and psychological adjustment (Thompson et al. 2003). Positive associations were found between meaning of life and positive affect and emotions. People reporting high levels of meaning experienced stronger positive affect (King et al. 2006) and less negative affect (Chamberlain and Zika 1988), depression and anxiety (Riichiro and Masahiko 2006). Yet, these results were obtained by using different measures of meaning in life, so it is difficult to draw a definite conclusion about relationships between meaning in life and SWB. King and Hicks (2012) point out that the correlations between meaning in life and positive affect range from .45 between positive affect and a composite of purpose in life and sense of coherence questionnaires to .80 for positive affect and sense of coherence.

Research on different facets of PWB shows that it is associated with more optimal human functioning and health benefits (Ryff and Singer 2008), achievement striving (Steger et al. 2008), global happiness (Ryff and Keyes 1995), and a stronger sense of coherence (Krok 2009). There were also found positive associations between meaning in life and PWB suggesting that a sense of purpose and significance is related to personal development and self-realization (Krok 2011). The results indicate that the components considered to be central to the concept of meaning, i.e. feeling that life matters, identifying a sense of purpose, and achieving an understanding of one’s life, hold direct implications for PWB.

## The Well-Being Benefits and Costs Associated with Meaning in Life

Although empirical studies have examined the relationships between meaning in life and well-being, little attention has been paid to the systematic study of the underlying meaning structures that are associated with well-being. Meaning in life is a multidimensional construct which is especially noticeable in the concept of personal meaning. Research conducted by Wong (1998) revealed that the total and domain scores of personal meaning were positively correlated with PWB, but not with physical well-being. Existing research implies that in the general population the domains of personal meaning that refer to the individual sphere are more strongly connected to internal aspects of well-being (inner and individual forms of activities and self-reflection), while the social-related domains are linked to external facets of well-being (relations with other people and the world) (Krok 2011). The domains represent an existential nature of meaning in life which is of great importance for the formation of adolescents’ identity and their pursuit of significant goals and values.

Furthermore, it is unknown how presence of meaning and search for meaning are related to different dimensions of subjective and psychological well-being. Empirical and theoretical work on meaning in life has provided strong evidence for maintaining a distinction between having meaning and searching for meaning (Steger 2009, 2012). The relations between presence of meaning and search for meaning have not been fully established and they require further investigation. Some individuals who are searching for meaning can struggle to establish some minimal level of presence of meaning in their lives, whereas others, already having a relatively high level of meaning, might be constantly striving to deepen their sense of purpose and significance and consider themselves to be in a life-long search for meaning (Steger et al. 2008).

Previous studies examining meaning in life focused mainly on adults and less attention has been paid to adolescents. However, the period of adolescence is associated with significant changes in achieving a sense of purpose and decision-making processes regarding meaning, values, and goals. Finding meaning in life and establishing a coherent philosophy of life are the critical issues underlying developmental processes during the period of adolescence (Erikson 1982; Steger et al. 2012). Recent research has revealed that a sense of life meaning has positive links with psychological and academic adjustment (Kiang and Fuligni 2010), and is a protective factor against health risk behaviours and poor psychological health among adolescents (Brassai et al. 2011). Meaning in life and optimism are also significantly associated with multidimensional life satisfaction and the multidimensional structure of psychosocial problems among adolescents (Ho et al. 2010).

Steger and his collaborators (Steger et al. 2009) point to noticeable variations in meaning in life among four life stage groups: emerging adulthood, young adulthood, middle-age adulthood, and older adulthood. Individuals at later life stages (middle-age adulthood and older adulthood) generally report a greater presence of meaning in their lives, whereas those at earlier life stages (emerging adulthood and young adulthood) tend to express higher levels of searching for meaning. Presence of meaning has similar relations to well-being across life stages, whereas search for meaning is more strongly linked with well-being deficits at later life stages. Among adults, presence of meaning in life is linked to desirable psychological outcomes such as life satisfaction, whereas the reported search for meaning is often linked to undesirable ones such as depression (Steger et al. 2006, 2009). However, search for meaning is positively associated with greater life satisfaction, more happiness, and less depression among those who already had substantial meaning in their life (Park et al. 2010).

These results suggest that the search for what makes life meaningful can be either productive or counter-productive for well-being, depending on the level of presence of meaning in life obtained by individuals. It is also not fully known how domains of personal meaning are related to dimensions of subjective and psychological well-being. Further investigation is needed to clarify our understanding of the search for and presence of life meaning and their relationships to well-being. It is especially a vital topic in research on adolescents who are a rather under-investigated group in this field.

The purpose of the present study was twofold. Firstly, it attempted to examine the relationships between presence of meaning in life (personal meaning) and dimensions of subjective and PWB in a sample of late adolescents from Poland (Study 1). Secondly, it aimed at examining the relationships between the presence of and search for meaning dimensions and subjective and PWB outcomes among Polish late adolescents (Study 2). In the present study, it was hypothesized that personal meaning and presence of meaning would have an overall positive relationship with well-being, while search for meaning would have negative relationships. However, search for meaning would be positively associated with well-being among individuals who had already obtained substantial meaning in life. In other words, individuals characterized by a relatively high level of presence of meaning and search for meaning would have the highest level of well-being. The results should determine whether young people with diverse levels of the presence and search dimensions differ in subjective and psychological well-being.

## Study 1

Study 1 focused on analysing the relationships between presence of meaning in life and subjective and psychological well-being. The concept of personal meaning was used as it accurately reflects presence of meaning in life and occupies a prominent place in research on meaning and well-being (King and Hicks 2012; McDonald et al. 2012). The study focused on the following hypotheses: (1) There will be positive associations between personal meaning and subjective and PWB measures; (2) The domains of personal meaning will be associated more strongly with PWB than with SWB.

## Method

### Participants and Procedure

One hundred and 95 participants (98 women and 97 men) participated in this study. They completed a questionnaire packet consisting of four methods. Ages ranged from 16 to 20 years, with a mean age of 18.69 years (*SD* = 1.12). There were no statistically significant differences in terms of age between women and men. The data were collected during classes in colleges in southern parts of Poland, aiming to cover a wide range of college characteristics for a representative sample of Polish late adolescents. All the participants were full-time college students who were not employed. They were invited to participate in the study on a voluntary and anonymous basis. Informed consent was obtained from the students and college authorities provided agreement to have the author conduct research.

#### Measures

The following four questionnaires were used to assess meaning in life and subjective and psychological well-being.

#### Meaning in Life

The Personal Meaning Profile (Wong 1998) evaluates personal meaning which is viewed as “comprising an ideally meaningful life.” This approach reveals people’s idealized prototypical structure of a meaningful life, relatively affected by their current life situations. The scale gauges the level of meaning reported by respondents in seven domains: achievement (“I strive to achieve my goals”), relationship (“I relate well to others”), religion (“I seek to do God’s will”), self-transcendence (“I have a purpose and direction in life”), self-acceptance (“I accept my limitations”), intimacy (“I have a good family life”), and fair treatment (“I am treated fairly by others”). The questionnaire contains 57 items evaluated on a 7-point Likert scale. A total score can also be calculated to reflect overall levels of personal meaning. The Cronbach alpha coefficients for the present study were .82 for the total score, and from .72 to .91 for the subscales.

#### Subjective Well-Being (SWB)

Two scales measuring cognitive and affective dimensions were used. The Satisfaction With Life Scale (SWLS; Diener et al. 1985) is a widely used and well-validated measure which can be applied to assess the cognitive dimension of SWB. The scale assesses the degree to which individuals are satisfied with their lives. It consists of five items rated from 1 (*absolutely untrue*) to 7 (*absolutely true*). Higher scores indicate greater satisfaction with life. The Cronbach alpha coefficient for the present study was .85.

The Positive and Negative Affect Schedule (PANAS-X; Watson and Clark 1991) assesses activated positive and negative affect, as well as 11 more specific affects (fear, hostility, guilt, sadness, joviality, self-assurance, attentiveness, shyness, fatigue, serenity, and surprise). It can be used to evaluate the affective component of SWB. In total, the PANAS-X has 60 items which are measured using a 5-point scale (1 = very slightly or not at all to 5 = extremely). In the current research, we only used positive and negative affect subscales. The Cronbach alpha coefficients for the present study were .85 for positive affect and .87 for negative affect.

#### Psychological Well-Being (PWB)

The PWB scale is a 42-item questionnaire that evaluates the level of individuals’ development and self-realization (Ryff and Keyes 1995). It comprises six scales: autonomy, environmental mastery, personal growth, positive relations with others, purpose in life, and self-acceptance. Each scale consists of seven items, with a mix of positive and negative items. On a scale from 1 to 6, respondents indicated whether they agreed or disagreed strongly, moderately, or slightly that an item described how they thought and felt. The Cronbach alpha coefficients for the present study ranged from .75 to .88.

## Results and Discussion

As an initial examination of the predicted relations, correlations were computed among personal meaning, life satisfaction, positive affect, negative affect, and PWB. The results are presented in Table 1.

**Table 1:** Correlations among personal meaning, life satisfaction, affect, and PWB dimensions

	Personal meaning							
	ACH	REL	RELI	ST	SA	INT	FT	PMP
Life satisfaction	.42***	.41***	.25***	.38***	.39***	.37***	.44***	.51***
Positive affect	.48***	.34***	.14	.38***	.25***	.10	.34***	.37***
Negative affect	− .18*	− .19**	.03	− .09	− .23**	− .19**	− .03	− .16*
Autonomy	.31***	.25***	.20**	.21**	.25***	.10	.20**	.29***
Environmental mastery	.24***	.08	.23**	.20**	.14	− .08	.09	.17*
Personal growth	.13	.09	.24***	.22**	.05	− .11	.15*	.15*
Positive relations with others	.12	.18*	.25***	.23**	.05	.03	.11	.19**
Purpose in life	.28***	.19**	.19**	.33***	.15*	.00	.24***	.26***
Self-acceptance	.31***	.19**	.19**	.25***	.16*	.10	.18*	.26***
PWB	.30***	.21**	.28***	.31***	.17*	.01	.21**	.28***

Personal meaning profile: *ACH* achievement, *REL* relationship, *RELI* religion, *ST* self-transcendence, *SA* self-acceptance, *INT* intimacy, *FT* fair treatment, *PMP* total personal meaning, *PWB* total psychological well-being.

***p < .001; **p < .01; *p < .05

The total personal meaning score was positively correlated with life satisfaction, positive affect, total PWB, and all its dimensions. In contrast, negative correlations were found between total personal meaning and negative affect. As regards the correlation analysis for particular dimensions of personal meaning, all of them were positively related to life satisfaction, and all but religion and intimacy were correlated with positive affect. Four dimensions of personal meaning, achievement, relationship, self-acceptance, and intimacy, were negatively associated with negative affect. Positive correlations were found between total PWB and all the dimensions of personal meaning except intimacy. In addition, intimacy showed no significant correlations with the dimensions of PWB.

In order to examine the relative contribution of personal meaning to subjective and PWB, a stepwise regression analysis was conducted (Table 2). The predictors were personal meaning dimensions and gender, while the dependent variables were subjective and psychological well-being.

**Table 2:** Stepwise regression statistics for subjective and PWB scales on dimensions of the personal meaning profile

	β	*t*	*p*
Life satisfaction: *R* = .56; *R* ^*2*^ = .32; *F*(3,191) = 17.18; *p* < .001			
Fair treatment	.34	4.67	.000
Intimacy	.21	2.98	.003
Achievement	.17	2.03	.043
Positive affect: *R* = .54; *R* ^*2*^ = .20; *F*(2,192) = 12.65; *p* < .001			
Achievement	.48	5.28	.000
Fair treatment	.25	3.21	.005
Gender	.20	2.89	.002
Negative affect: *R* = .21; *R* ^*2*^ = .09; *F*(1,193) = 4.75; *p* < .05			
Self-acceptance	− .20	− 2.39	.019
Psychological well-being: *R* = .36; *R* ^*2*^ = .13; *F*(2,192) = 6.99; *p* < .001			
Achievement	.20	1.99	.044
Relationship	.17	1.97	.049

In the first regression equation, the combined predictors accounted for a significant portion of variance (32%) in life satisfaction (*F* = 17.18; *p* < .001). The examination of standardized regression coefficients (β) revealed that fair treatment, intimacy, and achievement predicted higher levels of life satisfaction. In the regression equation for positive affect, the combined predictors accounted for 20% of variations (*F* = 12.65; *p* < .001). Three factors, achievement, fair treatment, and gender, predicted higher levels of positive affect. For negative affect, the only predictor was self-acceptance that accounted for 9% of variance (*F* = 4.75; *p* < .05). Self-acceptance was the only factor that predicted lower levels of negative affect. Finally, in the regression equation for PWB, the combined predictors accounted for 13% of variance (*F* = 6.99; *p* < .001). The results of the beta weights indicate that achievement and relationship predict higher levels of PWB.

In summary, the findings of Study 1 suggest that personal meaning has strong relations with SWB and PWB. It is clearly observable in both total scores and particular dimensions. The results confirmed the first hypothesis of positive associations between the personal meaning system and SWB and PWB. Personal meaning, as an individually constructed cognitive system that imbues life with significance and purpose, is strongly connected with a cognitive component of SWB, i.e. life satisfaction. The underlying assumption is that higher scores in the dimensions of personal meaning reflect more meaning in life. All the dimensions of personal meaning turned out to be positively correlated with life satisfaction. The regression equation for these factors was also characterized by the largest variance among all the equations computed at this stage of research. With regard to the affective components of SWB, personal meaning was positively related to positive affect and negatively associated with negative affect. This result corresponds with previous findings which indicated bidirectional associations, between meaning in life and emotions (King et al. 2006; Steger 2009). Existential experiences of fulfilment, which stem from a sense of significance and purpose appear to increase positive emotional states and reduce negative ones.

Personal meaning and most of its dimensions were positively related to PWB and its dimensions. Yet, there were differences in the associations of personal meaning with PWB and SWB. They were directly tested by assessing the significance of the difference between two correlation coefficients. There was a significant difference between the correlations of personal meaning with life satisfaction and personal meaning with PWB (*p* < .01). In contrast, there were no differences between the correlations of personal meaning with positive and negative affect and the correlation of personal meaning with PWB. The results suggest that personal meaning is associated more strongly only with the cognitive component of SWB, i.e. life satisfaction in comparison with PWB. It does not support the second hypothesis which assumed the opposite associations. This finding is rather puzzling as personal meaning reflects a sphere of existential strivings directed at achieving a meaningful life, which is based on a sense of purpose and significance. These qualities are also embedded in the concept of PWB that perceives life in terms of virtues and self-realization. One possible explanation for this effect may originate from an internal structure of personal meaning, which is primarily understood as a cognitive system (Wong 1998). It serves as a cognitive map that orients adolescents through their life experiences. Thus, the relationships between personal meaning and life satisfaction become somehow logical.

Study 1 focused on the presence of meaning in life and its relationships with well-being. It does not allow us to formulate any conclusions regarding situations in which people search for meaning. However, the theoretical field of research on meaning in life also comprises an understanding of the degree to which people are engaged in search for meaning and relations between presence of meaning and search for meaning. Therefore, in Study 2, we continued to examine the relations between both dimensions of meaning in life and their associations with subjective and psychological well-being.

## Study 2

The implications of Study 1 are limited by the use of personal meaning which only measures presence of meaning in life. Yet, recent research has argued for making a distinction between having meaning and searching for meaning (Steger 2009; Steger et al. 2006). Search for meaning in life denotes people’s desire and efforts to find more significance and purpose in their lives. The intensity of the presence and search dimensions in individuals’ mental states can lead to different associations for their well-being (Park et al. 2010).

Therefore, in Study 2, we sought to expand the findings by examining the relationships of different levels of presence of meaning and search for meaning with subjective and PWB among late adolescents. Consistent with previous studies, Study 2 focused on the following hypotheses: (1) Presence of meaning in life will positively correlate with subjective and psychological well-being, while search for meaning in life will have negative correlations; (2) Individuals characterized by a relatively high level of presence of meaning will have higher levels of subjective and PWB than those who search for meaning; (3) Individuals who have both meaning and search for meaning will have higher levels of subjective and PWB that those who have only meaning or search for meaning, respectively.

## Method

### Participants and Procedure

The participants were one hundred and eighty-nine participants (96 women and 93 men) who were randomly recruited in colleges located in southern parts of Poland. They were full-time college students who were not employed. Their age ranged from 16 to 20 years, with a mean age of 18.44 years (*SD* = 1.17). They were of a representative sample of Polish late adolescents in terms of social status, gender and age. The participants were given a questionnaire packet consisting of four methods and asked to fill them in during their classes. There were no statistically significant differences in terms of age between women and men. Informed consent was obtained from the students and college authorities provided agreement to have the author conduct research.

### Measures

Four questionnaires were administered: The Meaning in Life Questionnaire (MLQ), The Satisfaction with Life Scale (SWLS), The Positive and Negative Affect Schedule (PANAS-X), and The Psychological Well-Being scale (PWB). The last three scales were already presented in Study 1.

The Meaning in Life Questionnaire (MLQ) measures two dimensions of meaning in life: presence and search (Steger et al. 2006). It consists of 10 items rated on a 7-point scale from 1 (absolutely untrue) to 7 (absolutely true). The presence subscale evaluates the extent to which participants perceive their lives as meaningful (e.g. “I understand my life’s meaning” and “My life has no clear purpose”). The search subscale measures the extent to which respondents are actively seeking meaning or purpose in their lives (e.g. “I am searching for meaning in my life” and “I am looking for something that makes my life feel meaningful”). The Cronbach alpha coefficients for the subscales were .92 and .91, respectively.

## Results and Discussion

First, the correlations between the meaning in life scales and the subjective and PWB measures were examined (see Table 3).

**Table 3:** Correlations among presence of meaning, search for meaning, life satisfaction, positive affect, negative affect, and PWB dimensions

	Presence of meaning	Search for meaning
Life satisfaction	.46***	− .22**
Positive affect	.32***	− .04
Negative affect	− .15*	.07
Autonomy	.21**	.16*
Environmental mastery	.28***	− .02
Personal growth	.17*	− .05
Positive relations with others	.16*	.08
Purpose in life	.29***	.07
Self-acceptance	.20**	.06
Total PWB	.25***	.10

***p < .001; **p < .01; *p < .05

Presence of meaning was positively associated with life satisfaction, positive affect, and PWB and negatively associated with negative affect. However, search for meaning was only negatively correlated with life satisfaction and positively associated with autonomy.

Next, cluster analysis was used to explore the different patterns of people’s experiences of having and searching for meaning measured by the MLQ subscales. This analysis was selected to enable the identification of different styles of meaning in life. The two subscale measures (i.e. presence and search) were used as grouping variables. We conducted a non-hierarchical *k*-means cluster analysis, specifying a three-cluster solution.

In order to facilitate the interpretation of the meaning in life styles, scores on each MLQ subscale for each group with reference to the overall mean and standard deviation of the subscale were described. Interpretive criteria are based on dividing the total sample distribution on each subscale into threes. Table 4 presents means, standard deviations, and ANOVA results for three cluster groups representing the three meaning in life styles.

**Table 4:** Means, standard deviations, and the ANOVA results for three styles of meaning in life

	Search style (SS)		Presence style (PS)		Presence and search style (PSS)		*F*	*p*
	*M*	*SD*	*M*	*SD*	*M*	*SD*		
Presence of meaning	3.56	0.63	5.54	0.81	5.33	0.58	165.41	.000
Search for meaning	5.29	0.89	2.96	.67	5.21	0.61	104.52	.000

Cluster 1—search style (SS). Cluster 1 (*n* = 81) was characterized by low scores on presence of meaning, and high scores on search for meaning.

Cluster 2—presence style (PS). This cluster group (*n* = 49) demonstrated high scores on presence of meaning and low scores on search for meaning.

Cluster 3—presence and search style (PSS). This cluster group (*n* = 59) was characterized by high scores on presence of meaning as well as high scores on search for meaning.

One-way ANOVA was used to test for group differences in the proportion of individuals grouped into each cluster for both dimensions of meaning in life. The result for presence of meaning was significant, *F*(3, 185) = 165.41; *p* < .001 as well as for search for meaning, *F*(3, 185) = 104.52; *p* < .001. It confirms differences between the three samples in meaning in life characteristics.

Next, we compared the effects of the three styles of meaning in life, represented by cluster groups of individuals from the present sample, on relevant subjective and PWB variables and found several significant differences (Tables 5, 6).

**Table 5:** Means, standard deviations, and the ANOVA effects for SWB dimensions between styles of meaning in life

	Search style (SS)		Presence style (PS)		Presence and search style (PSS)		*F*	*p*	Tukey test
	*M*	*SD*	*M*	*SD*	*M*	*SD*			
Life satisfaction	3.72	.86	4.38	1.11	4.35	.91	11.08	.000	SS:PS**; SS: PSS**
Positive affect	3.17	.65	3.51	.54	3.54	.56	8.28	.000	SS:PS*; SS:PSS**
Negative affect	1.96	.64	1.76	.53	2.01	.65	2.28	.10	–

***p < .001; **p < .01; *p < .05

**Table 6:** Means, standard deviations, and the ANOVA effects for PWB dimensions between styles of meaning in life

	Search style (SS)		Presence style (PS)		Presence and search style (PSS)		*F*	*p*	Tukey test
	*M*	*SD*	*M*	*SD*	*M*	*SD*			
Autonomy	4.13	.53	4.18	.52	4.50	.64	6.81	.002	SS:PSS**; PS:PSS*
Environmental mastery	4.06	.58	3.87	.63	4.25	.72	4.41	.014	PS: PSS*
Personal growth	4.11	.54	3.93	.66	4.13	.71	1.47	.23	–
Positive relations with others	4.02	.52	3.98	.70	4.16	.60	1.11	.33	–
Purpose in life	3.91	.58	3.79	.60	4.14	.72	3.75	.025	PS:PSS*
Self-acceptance	4.12	.48	4.26	.58	4.42	.68	3.76	.024	SS:PSS**
Total score PWB	4.04	.38	4.01	.45	4.26	.57	4.47	.013	SS:PSS*; PS:PSS*

***p < .001; **p < .01; *p < .05

As regards SWB, the one-way ANOVA test revealed significant differences in the effects of the styles of meaning in life on life satisfaction *F*(3, 185) = 11.08; *p* < .001. Post hoc comparisons using the Tukey test pointed out that SS was significantly lower than PS and PSS. Similar results in the styles occurred for positive affect [*F*(3, 185) = 8.28; *p* < .001], with SS obtaining significantly lower scores than PS and PSS. In contrast, there were no significant effects of styles on negative affect scores [*F*(3, 185) = 2.28; *p* < .10].

Next, the one-way ANOVA was used to examine the effect of the styles of meaning in life on PWB dimensions (Table 6).

Results indicated statistically significant differences in the total PWB score for the styles [*F*(3, 185) = 4.47; *p* < .05]. Post hoc comparisons using the Tukey test demonstrated that individuals in PSS had higher levels of PWB that individuals in SS and PS.

For particular dimensions of PWB, there were significant differences in the styles for autonomy [*F*(3, 185) = 6.81; *p* < .001]. Post hoc comparisons using the Tukey test indicated that the group with presence and SS had higher levels of self-determination and independence than the groups with SS and presence style. Significant differences in the cluster groups were noted for environmental mastery [*F*(3, 185) = 4.41; *p* < .05], in which individuals in presence and SS experienced a stronger sense of mastery and competence in managing the environment than individuals in presence style. No significant differences were found for personal growth and positive relations with others [*F*(3, 185) = 1.47; *p* = .23; *F*(3, 185) = 1.11; *p* = .33, respectively]. There were significant effects of the styles on purpose in life [*F*(3, 185) = 3.75; *p* < .05]. Post hoc comparisons using the Tukey test revealed that the group with presence and SS had higher levels of purpose and goals that the group with presence style. Finally, the one-way ANOVA test showed significant differences in the styles score for self-acceptance [*F*(3, 185) = 3.76; *p* < .05]. The Tukey test results indicated that individuals in presence and SS had more positive attitudes toward the self than individuals in SS.

The results obtained in Study 2 complement the previous ones. Positive correlations were found between presence of meaning and life satisfaction and positive affect, while negative associations occurred between presence of meaning and negative affect. The underlying assumption is that higher scores in presence of meaning reflect higher levels of meaning in life, while a higher score in search for meaning represents more active seeking meaning in one’s life. The results point to positive relationships between presence of meaning in life and SWB among late adolescents. The same pattern of relations was found between presence of meaning and PWB. Both the total score and all the dimensions were positively associated with presence of meaning. With regard to the relationships between search for meaning and subjective and PWB, the only negative correlation was found for life satisfaction. These findings allow us to confirm the first hypothesis only with regard to positive associations between presence of meaning and well-being.

ANOVA tests revealed that late adolescents in PS had higher levels of life satisfaction and positive affect than their peers in SS. In contrast, there were no significant differences in PWB dimensions between PS and SS. Therefore, the second hypothesis was confirmed with regard to SWB. The highest level of subjective and PWB was obtained by late adolescents in presence and SS whose scores significantly differed from the scores of their peers in PS and SS in most dimensions of well-being. These results enable us to verify the third hypothesis which predicted this pattern of relations.

## General Discussion

The primary objective of this study was to investigate the relationships between different dimensions of meaning in life and subjective and PWB among late adolescents. Having a sense of meaning and purpose in life seems crucial for well-being, from both the hedonistic and eudaimonic perspectives. The present investigation is an extension of earlier studies in which researchers examined separately presence of meaning in life and search for meaning in life (Steger and Frazier 2005; Zika and Chamberlain 1992) and provides a more advanced quantitative modelling of the complex relationships between the dimensions of meaning in life and well-being among late adolescents than existing research (Głaz 2013; Park et al. 2010). The major contribution of the present study is to demonstrate that search for meaning has positive associations with subjective and PWB among those late adolescents who already have substantial meaning in life. It may be also worth noting that the results were drawn from Polish samples, which differ from North America and Western Europe.

Late adolescence is a developmental stage in which individuals struggle to make sense of the world and themselves, and to achieve an integrated view of goals and values. In this sense, search for meaning is conducive to the adolescent identity formation as young people strive to understand more fully their life and establish overarching aims which are clearly distinguishable from previous developmental stages (Boyd and Bee 2012; Reker 2005). The findings obtained in the current study revealed that meaning in life understood existentially and expressed in personal and interpersonal domains is positively associated with subjective and PWB. Adolescents, who can successfully draw meaning from such sources as personal achievements, intimate and fulfilling relationships, religious values, self-acceptance, and a non-discriminative community, are able to increase their life satisfaction and positive emotions, and to achieve higher levels of value-related growth and self-realization. The primary sources of meaning are concerned with personal significance and individuals’ core values, which are vital to a young person’s identity and self-esteem.

These results are consistent with previous findings in which meaning in life was a valuable indicator of positive functioning (Steger 2012; Wong 2012). They also extend previous studies regarding meaning in life and well-being (King et al. 2006; Zika and Chamberlain 1992) by demonstrating that intrapersonal and interpersonal sources of meaning in life are associated with hedonic and eudaimonic well-being among late adolescents. Meaning in this sense enables young people to interpret and organize their daily experience, identify elements that are important to them, and effectively direct their efforts to resolve potential problems.

Study 2 showed that understanding the role that search and presence play in well-being among late adolescents requires taking into account the conditions in which people found meaning and yet actively search for meaning in their lives. Cluster analysis enabled us to identify three distinct cluster groups, which represent an integrative and nuanced depiction of the adolescents’ experiences of meaning in life. The three styles of meaning in life denote different approaches to the sphere of purpose and significance which is an important factor needed for young people to reach maturity and form an integrated view of life.

These individuals who were in presence and SS had higher levels of subjective and PWB that those in SS or presence style. This indicates that when young people already have meaning, they have a solid foundation that allows the search for further meaning to be a beneficial and productive process for increasing well-being. In contrast, when adolescents have not attained a satisfactory level of meaning in life, their search for meaning can be problematic and frustrating.

This finding is important for future research as it suggests that search for meaning does not have to necessarily be viewed in negative terms as a factor detrimental to life satisfaction. Although among adults search for meaning is often linked to undesirable psychological outcomes such as depression and lower life satisfaction (Steger et al. 2006, 2009), it can play a positive role for those adolescents who already have meaning in their life. This interpretation is consistent with the findings obtained by Park et al. (2010) who demonstrated that search for meaning was positively associated with greater life satisfaction, more happiness, and less depression among adults with a relatively high level of meaning in life. Therefore, search for meaning might play a larger and more gratifying role among younger populations than older populations. Research on adolescents also suggests that search for meaning may be conducive to the identity exploration in developmental crises that are a normal, healthy part of maturation (Steger et al. 2009).

Another important finding was that late adolescents in PS had higher levels of SWB than their peers in SS. In contrast, there were no significant differences in PWB between PS and SS. These results are somehow unexpected and intriguing. Why does presence of meaning in relation to search for meaning matter in SWB, but not in PWB? The answer to this question may lie in the conceptual structures of both types of well-being and their relevance for adolescents.

SWB, being based on a hedonic approach, stresses subjective experiences of pleasure and satisfaction, whereas PWB, which is grounded in a eudaimonic perspective, focuses on self-realization, growth, and goal pursuits (Diener et al. 2009; Ryff and Singer 2008). Given developmental characteristics in late adolescence, we should expect that young people in forming their self-concepts and social relationships are more likely to concentrate on attaining pleasure and life satisfaction than to deliberately focus on self-realization and personal growth. As a consequence, presence of meaning will be more central to constructing a sense of SWB than search for meaning. In addition, search for meaning is usually related to tendencies to engage with negative thinking about one’s self and past (Steger et al. 2008). This appear to be detrimental to experiencing satisfaction and positive emotions, and thus, it should be eliminated from the mental activities directed at enhancing SWB.

There are several limitations in this study which future research could address. First, the present study was a cross-sectional survey, which severely curtails inferences about causal relations between the dimensions of meaning in life and the types of well-being. Future longitudinal studies will provide additional information about the developmental trajectory of presence and search. Experimental methods might also examine whether adolescents searching for meaning respond differently to changes in subjective and psychological well-being. Second, our participants were college students, and though most young people at that age attend high school institutions, some of them have already left school and found work. The work-related factor could modify one’s sense of significance and overarching aims, which in turn would influence domains of subjective and psychological well-being. Thus, our results may not be generalized to the late adolescents who have a permanent job. Third, our measurement of meaning in life and SWB was restricted to a single point in time which does not account for potential fluctuations. Cognitive and emotional reactions concerning meaning in life and happiness vary in response to life events, and other ways of measuring people’s quest for meaning and well-being may reveal different relations.

Despite the aforementioned limitations, the current research extends the previous findings on meaning and well-being by examining the relationships between personal meaning, presence and search for life meaning and well-being outcomes in late adolescents. Across two studies, our results clearly indicate that experiencing a sense of meaning and purpose in life is important for both subjective and psychological well-being. Late adolescents construe meaning on a basis of intrapersonal and interpersonal factors in order to attain life satisfaction and enhance growth and self-realization. The interaction between presence of meaning and search for meaning appears crucial to understanding young people’s well-being judgments.

Search for meaning has positive associations with subjective and PWB among those late adolescents who already have substantial meaning in life. While personal meaning and presence of meaning have positive associations with both types of well-being, search for meaning is positively linked with well-being only among those adolescents who already had substantial meaning in their life. Further examination of search for meaning in life is warranted within the domain of well-being research as searching for meaning plays an important role in attaining happiness and achieving optimal levels of positive functioning among late adolescents.
